# Further delineation of phenotype and genotype of Kenny–Caffey syndrome type 2 (phenotype and genotype of KCS type 2)

**DOI:** 10.1002/mgg3.2433

**Published:** 2024-04-09

**Authors:** Xuefei Chen, Chaochun Zou

**Affiliations:** ^1^ Department of Endocrinology, Children's Hospital Zhejiang University School of Medicine Hangzhou China

**Keywords:** FAM111A, genotype, hypocalcemia, hypoparathyroidism, Kenny–Caffey syndrome type 2, phenotype, short stature

## Abstract

**Background:**

Kenny–Caffey syndrome type 2 (KCS2) is an extremely rare inherited disorder characterized by proportionate short stature, skeletal defects, ocular and dental abnormalities, and transient hypocalcemia. It is caused by variants in *FAM111A* gene. Diagnosis of KCS2 can be challenging because of its similarities to other syndromes, the absence of clear hallmarks and the deficient number of genetically confirmed cases. Here, we aimed to further delineate and summarize the genotype and phenotype of KCS2, in order to get a better understanding of this rare disorder, and promote early diagnosis and intervention.

**Methods:**

We present clinical and genetic characteristics of eight newly affected individuals with KCS2 from six families, including one family with three individuals found to be a father‐to‐daughter transmission, adding to the limited literature*.* Furthermore, we performed a review of genetically confirmed KCS2 cases in PubMed, MEDLINE and CNKI databases.

**Results:**

There were six females and two males in our cohort. All the patients presented with short stature (100.0%). Clinical manifestations included ocular defects such as hypermetropia (5/8), dental problems such as defective dentition (3/8) and dental caries (3/8), skeletal and brain anomalies such as delayed closure of anterior fontanelle (6/8), cerebral calcification (3/8), cortical thickening (3/8) and medullary stenosis (4/8) of tubular bones. Endocrinologic abnormalities included hypoparathyroidism (5/8) and hypocalcemia (3/8). One male patient had micropenis and microorchidism. All cases harboured missense variants of *FAM111A*, and nucleotides c.1706 arose as a mutational hotspot, with seven individuals harbouring a c.1706G>A (p.Arg569His) variant, and one child harbouring a c.1531T>C (p.Tyr511His) variant. Literature review yielded a total of 46 patients from 20 papers. Data analysis showed that short stature, hypoparathyroidism and hypocalcemia, ocular and dental defects, skeletal features including cortical thickening and medullary stenosis of tubular bones, and seizures/spasms were present in more than 70% of the reported KCS2 cases.

**Conclusion:**

We provide detailed characteristics of the largest KCS2 group in China and present the first genetically confirmed instance of father‐to‐daughter transmission of KCS2. Our study confirms that Arg569His is the hot spot variant and summarizes the typical phenotypes of KCS2, which would help early diagnosis and intervention.

## Graphical abstract

We provided detailed characteristics of eight individuals with genetically confirmed KCS2, which has been the largest cohort of KCS2 so far in China. We present the first genetically confirmed instance of father‐to‐daughter transmission of KCS2, which demonstrates an equal sex ratio of affected individuals.

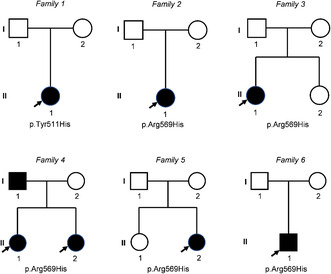

## 1 INTRODUCTION

Kenny–Caffey syndrome (KCS) (MIM 244460) is a rare condition characterized by severe proportionate short stature, cortical thickening and medullary stenosis of tubular bones, delayed closure of the anterior fontanelle, ocular and dental anomalies, and variable hypocalcemia due to hypoparathyroidism (Caffey, 1967; Fanconi et al., 1986; Kenny & Linarelli, 1966). KCS was first described as a rare genetic disorder by Kenny and Linarelli in 1966 (Kenny & Linarelli, 1966). Later, Caffey reported the radiologic findings to correspond to the disease in 1967 (Caffey, 1967). It is classified into two types according to clinical features and inheritance pattern (Franceschini et al., 1992). Whereas KCS type 1 (KCS1) (MIM 244460) is caused by mutations in tubulin folding cofactor E (TBCE) gene (Fanconi et al., 1986; Sabry et al., 1998, 1999), KCS type 2 (KCS2) (MIM 127000) is resulted from mutations in family with sequence similarity 111 member A (FAM111A) gene (NM_001142519.1) (Unger et al., 2013). The *TBCE* gene encodes a molecular chaperone required for proper folding of α‐tubulin and its heterodimerization with β‐tubulin (Parvari et al., 2002). The *FAM111A* gene encodes a protein comprising 611 amino acids playing a role in DNA replication (Kojima et al., 2020) and viral defense (Fine et al., 2012).

The autosomal dominant form of KCS (KCS2) is distinguished from the autosomal recessive form of KCS (KCS1), by the absence of mental and prenatal growth retardation (Isojima et al., 2014; Parvari et al., 2002). The causative gene of KCS2, *FAM111A* gene, was first identified by the Swiss group (Unger et al., 2013). Delayed closure of anterior fontanelle, as well as cortical thickening and medullary stenosis of tubular bones, was more common in KCS2 than KCS1 (Schigt et al., 2023). On the contrary, intellectual disability, microcephaly and intrauterine growth retardation were much more common in KCS1 (Parvari et al., 2002). Even so, differential diagnosis can be challenging due to numerous overlapping symptoms in KCS1 and KCS2. Postnatal growth retardation, cerebral calcification, seizures, eye and dental abnormalities, low parathyroid hormone and hypocalcemia can occur in both KCS types (Isojima et al., 2014; Schigt et al., 2023).

Heterozygous mutations in *FAM111A* gene have been found in patients with osteocraniostenosis (OCS, also known as gracile bone dysplasia, MIM 602361) as well. OCS is a severe, usually lethal condition characterized by gracile bones with thin diaphyses, a cloverleaf‐shaped skull and splenic hypo/aplasia (Verloes et al., 1994). OCS and KCS2 share some common features, including frontal bossing, triangular face, microphthalmia, thin long bones and hypocalcemia (Elliott et al., 2006; Rosato et al., 2022; Verloes et al., 1994). Unger et al. (2013) speculated that KCS2 and OCS might be allelic disorders for monoallelic variants in *FAM111A* in all KCS2 and OCS patients. While there is phenotypic overlap between the two conditions, OCS is more severe and typically leads to perinatal death (Eren et al., 2021; Unger et al., 2013). It is urgent to expend the phenotypes and establish typical features of KCS2, so as to improve the awareness and ability of physicians to undertake accurate diagnosis and appropriate investigations.

KCS2 is an extremely rare condition. In a study involving 37 pediatric patients with primary hypoparathyroidism, one case with KCS2 was identified (Kim et al., 2015). In a large group of Chinese patients with childhood‐onset hypoparathyroidism, only one out of 173 patients was reported with KCS2 (Wang et al., 2019). Until now, a total of six patients from five papers had been reported in China (Cheng et al., 2021; Wang et al., 2019; Yuan et al., 2023), including a pair of monozygotic twins (Cheng et al., 2021). In this study, we recruited eight Chinese individuals with heterozygous pathogenic variants in *FAM111A* from six families, adding to the phenotypic spectrum of KCS2. To the best of our knowledge, this is the largest cohort of genetically proven KCS2 cases in China. Moreover, we performed a literature review of all genetically confirmed KCS2 in order to facilitate a better understanding of clinical features and molecular mechanisms of this condition, aiding diagnosis and early intervention.

## 2 MATERIALS AND METHODS

### 2.1 Ethical compliance

The study was performed with the approval of the Medical Ethical Committee of the Children's Hospital of Zhejiang University School of Medicine (2023‐IRB‐0166‐P‐01) and conducted in accordance with the Declaration of Helsinki. The patients' parents have given written informed consent for the use of clinical information in this study.

### 2.2 Subjects and genetic investigations

We identified eight patients from six families with pathogenic variants in *FAM111A* gene. After obtaining the informed consent from patients' parents, genomic DNA was extracted from peripheral blood samples of the patients and their parents. Whole‐exome sequencing was performed on Illumina HiSeq X ten platform. Detected *FAM111A* variants were subsequently confirmed by Sanger sequencing. Patients were diagnosed based on clinical characteristics, laboratory measurements and genetic testing.

### 2.3 Literature review

In order to get a comprehensive overview of phenotypic and genotypic spectrum in confirmed cases of KCS2, a search was performed of the PubMed, MEDLINE, and CNKI databases from 2013 (the year KCS2 was first reported) to 2023. The search keywords included “Kenny–Caffey syndrome” or “Kenny–Caffey syndrome type 2” or “FAM111A”. Demographical data, genetic results, main clinical symptoms, dysmorphisms, laboratory measurements, the first author's name and year of publication were extracted.

### 2.4 Statistical analysis

For assessment of the correlation of genotype and phenotype, two groups of KCS2 patients were assessed: (1) patients with a c.1706G>A (p.Arg569His) variant (*N* = 32) and (2) patients with missense or deletion mutations other than c.1706G>A (*N* = 14). Continuous variables were summarized using mean and ranges, and categorical variables using frequencies and percentages. Percentages were calculated by dividing the number of patients with positive symptoms by the number of patients for whom information has been provided, multiplied by 100. Differences between the two groups for continuous variables were evaluated using the Student's *t* test. Differences between the two groups on categorical variables were evaluated using the Chi‐square test. A *p*‐value <0.05 was considered statistically significant. Statistical analysis was performed by SPSS software, version 20.0.

## 3 RESULTS

**FIGURE 1:**
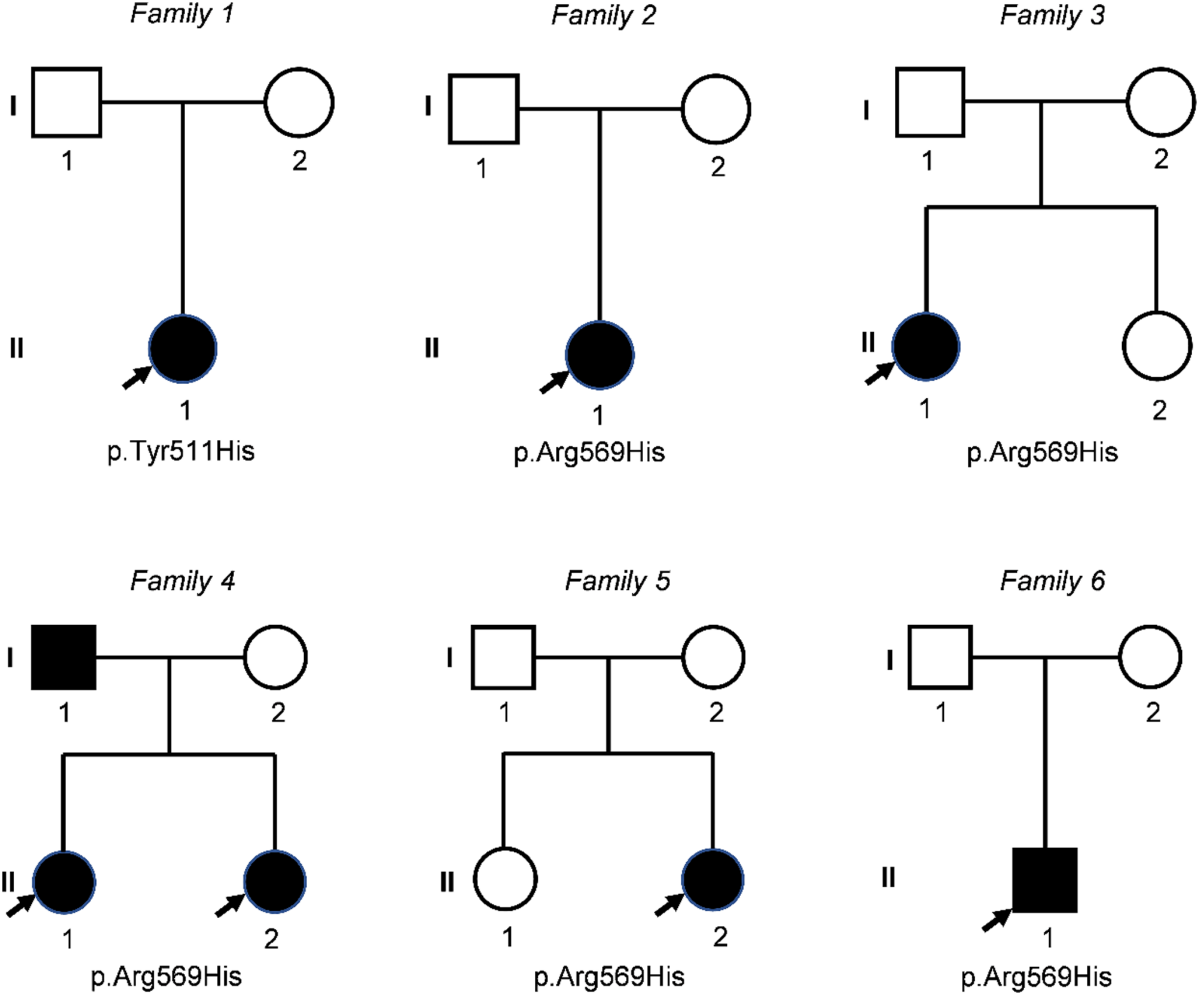
Family pedigrees of KCS2 patients in our cohort. In each pedigree, a black symbol represents the patient, a square indicates a male, and a circle shows a female. Probands are indicated with arrows.

The clinical characteristics and genetic results of our cohort are presented in Table 1, and the pedigrees are indicated in Figure 1. The main clinical features and molecular findings of all genetically confirmed patients are summarized in Table 2.

**TABLE 1:** Clinical features and genetic characteristics of our cases with KCS2.

Patient	Gender	Age	*FAM111A* variant	Inheritance	Length/height	Facial features	Ocular defects	Dental problems	Skeletal features and imaging	Hypocalcemia	Seizures	Endocrinological data (Ca, P, iPTH)			Other features
1	F	2 years and 4 months	c.1531T>C (p.Tyr511His)	de novo	−3.21 SDs	Prominent forehead, depressed nasal bridge	Not affected	Defective dentition, microdontia	Open anterior fontanelle, calcification in the basal ganglia, cortical thickening and medullary stenosis of long bones	Yes	Yes	1.9	2.85	4.8	Liver dysfunction, anemia
2	F	6 years	c.1706G>A (p.Arg569His)	de novo	−5.86 SDs	Prominent forehead, small eyes, flat nasal root	High hyperopia, amblyopia, tortuous retinal vessels	Dental caries, oligodontia, delayed dental eruption	Open anterior fontanelle	No	No	2.52	1.88	<2.88	GH deficiency, GH therapy
3	F	5 years and 9 months	c.1706G>A (p.Arg569His)	de novo	−3.99 SDs	Small eyes, high arched palate, micrognathia	Hypermetropia, amblyopia, pseudopapilledema	Dental caries, partial loss of dental germ	Open anterior fontanelle, cerebral calcification, cortical thickening and medullary stenosis of long bones	No	No	2.47	1.69	4.95	Not presented
4 (Twin 1)	F	6.5 years	c.1706G>A (p.Arg569His)	Paternally inherited	−5.25 SDs	Prominent forehead, small eyes, depressed nasal bridge, low‐set ears, micrognathia	Hypermetropia, astigmatism	Dental caries	Open anterior fontanelle, slender diaphyses	Yes	No	0.4–0.8	NA	NA	IUGR
5 (Twin 2)	F	6.5 years	c.1706G>A (p.Arg569His)	Paternally inherited	−7.91 SDs	Prominent forehead, small eyes, depressed nasal bridge, low‐set ears, micrognathia	Hypermetropia, astigmatism, pseudopapilledema	Not affected	Open anterior fontanelle	No	No	–	–	NA	IUGR, ADHD, liver dysfunction
6 (Father)	M	NA	c.1706G>A (p.Arg569His)	de novo?	−4.39 SDs	Not presented	Hypermetropia, astigmatism	Not affected	NA	No	No	–	–	–	Not presented
7	F	11 months	c.1706G>A (p.Arg569His)	de novo	−2.70 SDs	Frontal bossing, small eyes, depressed nasal bridge	Not affected	Not affected	Calcification in the basal ganglia, cortical thickening and medullary stenosis of long bones	Yes	Yes	1.26	3.67	13.5	Not presented
8	M	4 years	c.1706G>A (p.Arg569His)	de novo	−4.44 SDs	NA	Not affected	Not affected	Open anterior fontanelle, slender diaphysis, medullary stenosis of tubular bones	No	No	2.32	1.92	8.0	Micropenis, microorchidism

Abbreviations: −, normal but without specific data; ADHD, attention‐deficit hyperactivity disorder; Ca, serum calcium (mmol/L); GH, growth hormone; iPTH, intact parathyroid hormone (pg/mL); IUGR, intrauterine growth retardation; NA, not available; P, phosphorus (mmol/L); SD, standard deviation.

**TABLE 2:** Summary table of clinical features and molecular findings in patients with KCS2.

No.	Gender	Age	*FAM111A* variant	Inheritance	Short stature	ID	Seizures/spasms	Prominent forehead	Small eyes	Depressed nasal bridge	Micrognathia	Hypermetropia	(Pseudo‐) Papilledema
1	F	40y	c.1706 G>A	de novo	Yes	No	NA	Yes	Yes			Yes	No
2	M	17y	c.1706 G>A	NA	Yes	No	NA	Yes	Yes			Yes	No
3	M	10y	c.1706 G>A	NA	Yes	No	NA	Yes	Yes			Yes	No
4	F	6m	c.1706 G>A	de novo	Yes	No	NA	Yes	Yes			No	No
5	M	7y	c.1531 T>C	NA	Yes	No	NA	Yes	Yes			No	No
6	F	2y	c.1706 G>A	de novo	Yes	No	NA	Yes		Yes	Yes	Yes	Yes
7	M	4y	c.1706 G>A	de novo	Yes	No	Yes	Yes		Yes	Yes	Yes	No
8	F	5y	c.1706 G>A	de novo	Yes	No	Yes	Yes			Yes	Yes	No
9	M	12y	c.1706 G>A	de novo	Yes	No	Yes	Yes			Yes	Yes	Yes
10	F	12y	c.1706 G>A	de novo	Yes	No	No	Yes		Yes		No	No
11	F	3y	c.1706 G>A	Maternally inherited	Yes	No	Yes	Yes	Yes			NA	NA
12	F	25y	c.1706 G>A	de novo	Yes	No	Yes	No				NA	NA
13	M	14d	NA (p. Cys485Phe)	de novo	NA	No	NA	Yes				NA	NA
14	F	6y	c.1622 C>A	de novo	Yes	No	Yes					No	No
15	NA	10y	c.1706 G>A	NA	NA	NA	NA	NA	NA	NA	NA	NA	NA
16	M	8m	c.1706 G>A	de novo	Yes	No	Yes	NA	NA	NA	NA	No	No
17	M	18y	c.1706 G>A	de novo	Yes	Yes	Yes	Yes	Yes			Yes	
18	M	5m	c.968 G>A	de novo	Yes	No	NA	Yes	Yes		Yes		
19	F	10y	c.1706 G>A	de novo	Yes	Yes	NA				Yes	Yes	
20	M	23y	c.1621 T>C	de novo	Yes	No	Yes	Yes	No	No	Yes	Yes	
21	M	23y	c.1621 T>C	de novo	Yes	No	Yes	Yes	No	No	Yes	Yes	
22	F	20w.g.	c.1685 A>C	de novo	NA	NA	NA	Yes		Yes		NA	NA
23	F	9y	c.1706 G>A	NA	Yes	No	Yes				Yes	Yes	
24	M	2m	c.976 T>A & c.1714_1716del	Paternally and maternally inherited	Yes	No	NA		Yes	Yes		NA	NA
25	F	7y	c.1706 G>A	NA	Yes	No	No					NA	NA
26	M	2.5m	c.1706 G>A	de novo	Yes	No	Yes	Yes	No	No	No	No	No
27	F	56y	c.1706 G>A	NA	Yes	No	NA			Yes		No	No
28	M	9y	c.1579 C>A	de novo	No	No	Yes	NA	NA	NA	NA	Yes	
29	M	9m	c.1706 G>A	de novo?	Yes	Yes	Yes	NA	NA	NA	NA	No	No
30	F	18y	c.1706 G>A	de novo	No	No	Yes	NA	NA	NA	NA	Yes	
31	F	20y	c.1706 G>A	AD?	NA	No	No	NA	NA	NA	NA	Yes	
32	M	66y	NA	de novo?	Yes	Yes	Yes	NA	NA	NA	NA	NA	NA
33	M	57y	c.1604 A>G	AD?	Yes	Yes	Yes	NA	NA	NA	NA	NA	NA
34	F	NA	c.1604 A>G	AD?	Yes	No	Yes	NA	NA	NA	NA		
35	F	59y	c.1604 A>G	AD?	Yes	NA	No	NA	NA	NA	NA		
36	M	10y	c.1706 G>A	de novo?	Yes	No	Yes	NA	NA	NA	NA	No	No
37	F	4y	c.1706 G>A	de novo?	Yes	No	Yes	Yes				Yes	Yes
38	F	18y	c.1706 G>A	de novo	Yes	No	Yes	Yes	No	Yes	Yes	Yes	No
39	F	2.3y	c.1531 T>C	de novo	Yes	No	Yes	Yes		Yes		No	No
40	F	6y	c.1706 G>A	de novo	Yes	No	No	Yes	Yes	Yes		Yes	No
41	F	5.7y	c.1706 G>A	de novo	Yes	No	No		Yes		Yes	Yes	Yes
42	F	6.5y	c.1706 G>A	Paternally inherited	Yes	No	No	Yes	Yes	Yes	Yes	Yes	Yes
43	F	6.5y	c.1706 G>A	Paternally inherited	Yes	No	No	Yes	Yes	Yes	Yes	Yes	No
44	M	NA	c.1706 G>A	de novo?	Yes	No	No	No	No	No	No	Yes	No
45	F	11m	c.1706 G>A	de novo	Yes	No	Yes	Yes	Yes	Yes	No	No	No
46	M	4y	c.1706 G>A	de novo	Yes	No	No	NA	NA	NA	NA	No	No
Sum	25F; 20M	20 w.g.‐66y	32/46 (69.6%) c.1706 G>A	31/39 (79.5%) de novo (?)	40/42 (95.2%)	5/43 (11.6%)	23/33 (69.7%)	25/34 (73.5%)	14/34 (41.2%)	12/34 (35.3%)	13/34 (38.2%)	22/37 (59.5%)	5/37 (13.5%)

No.	Defective dentition	Dental caries	Oligodontia	Delayed closure of anterior fontanelle	Cortical thickening	Medullary stenosis	Cerebral calcification	Hypoparathyroidism	Hypocalcemia	GH deficiency	GH therapy	Other abnormalities	Year	References
1	Yes	NA	Yes	NA	NA	NA	NA	NA	Yes	NA	NA	Basal craniosynostosis, high pitched voice	2013	Unger et al. (2013)
2	Yes	NA	NA	NA	Yes	Yes	NA	NA	Yes	NA	NA	NA	2013	Unger et al. (2013)
3	No	No	No	NA	NA	NA	NA	NA	NA	NA	NA	NA	2013	Unger et al. (2013)
4	No	No	No	NA	NA	NA	NA	NA	Yes	NA	NA	NA	2013	Unger et al. (2013)
5	Yes	NA	NA	Yes	NA	NA	NA	NA	NA	NA	NA	High pitched voice	2013	Unger et al. (2013)
6	NA	NA	NA	Yes	Yes	Yes	Yes	Yes	Yes	Yes	NA	Polysyndactyly, anemia	2014	Isojima et al. (2014)
7	NA	NA	NA	Yes	Yes	Yes	No	Yes	Yes	Yes	NA	Severe atopic dermatitis	2014	Isojima et al. (2014)
8	NA	NA	NA	Yes	Yes	Yes	Yes	Yes	Yes	NA	NA	Hypothalamic amenorrhea	2014	Isojima et al. (2014)
9	NA	NA	NA	Yes	Yes	Yes	Yes	Yes	Yes	NA	NA	Humoral immunodeficiency	2014	Isojima et al. (2014)
10	Yes	No	Yes	NA	Yes	Yes	NA	No	No	No	Yes	NA	2014	Guo et al. (2014)
11	Yes	Yes	NA	Yes	NA	Yes	NA	Yes	Yes	NA	NA	IUGR	2014	Nikkel et al. (2014)
12	NA	NA	Yes	Yes	Yes	Yes		Yes	Yes	NA	NA	Hypothyroidism	2014	Nikkel et al. (2014)
13	NA	NA	NA	Yes	Yes	NA	NA	Yes	NA	NA	NA	Died of sepsis at 5 months of age	2015	Kim et al. (2015)
14				Yes	Yes	Yes		Yes	Yes	No	Yes	Small hands and feet, triangular dysplastic nails	2017	Abraham et al. (2017)
15	Yes			Yes	Yes	Yes		Yes	NA	NA	NA	Eye abnormalities	2019	Wang et al. (2019)
16	No	No	No		Yes	Yes		Yes	Yes	NA	NA	NA	2019	Xu et al. (2019)
17		Yes	Yes		Yes	Yes	Yes	Yes	Yes	NA	NA	Micropenis, microorchidism, nephrocalcinosis	2020	Cavole et al. (2020)
18	NA	NA	NA	Yes				NA	Yes	NA	NA	Passed away at 20 months of age	2020	Turner et al. (2020)
19	No	No	No				Yes	Borderline	Yes	NA	Yes	NA	2020	Deconte et al. (2020)
20		Yes		Yes	Yes	Yes		Yes	Yes	NA	NA	Rigid spine, scoliosis, nephroncalcinosis, microorchidism	2021	Cheng et al. (2021)
21				Yes	Yes	Yes		Yes	Yes	NA	NA	Rigid spine, torticollis, nephrocalcinosis, microorchidism	2021	Cheng et al. (2021)
22	NA	NA	NA	NA	NA	NA	NA	NA	NA	NA	NA	Hypoplastic spleen, termination of pregnancy at 20 w.g.	2021	Müller et al. (2021)
23	NA	NA	NA		Yes	Yes		Borderline	Yes	NA	NA	NA	2021	Yerawar et al. (2021)
24	NA	NA	NA	NA	NA	NA	NA	Yes	Yes	NA	NA	Micropenis, microorchidism, died due to septicemia at 3.5 months of age	2021	Eren et al. (2021)
25	NA	NA	NA		Yes	Yes		No	No	NA	NA	Nanophthalmos	2021	Lang et al. (2021)
26	NA	NA	NA	NA	Yes	Yes	No	Yes	Yes	NA	NA	Micropenis, liver dysfunction	2021	Huang et al. (2021)
27		Yes		NA	Yes	Yes	Yes	Yes	Yes	NA	NA	Hyperuricemia, gout	2022	Ohmachi et al. (2022)
28	NA	NA	NA	Yes	Yes	Yes		Yes	Yes	NA	NA	CKD, recurrent severe infections	2023	Schigt et al. (2023)
29	NA	NA	NA	NA	NA	NA	NA	Yes	Yes	NA	NA	Hepatitis	2023	Schigt et al. (2023)
30			Yes	Yes	No	No		Yes	Yes	NA	NA	IUGR, one ovary missing	2023	Schigt et al. (2023)
31	Yes					Yes		Yes	Yes	NA	NA	CKD	2023	Schigt et al. (2023)
32	NA	NA	NA				Yes	Yes	Yes	NA	NA	CKD, died at 77 years due to kidney failure	2023	Schigt et al. (2023)
33	NA	NA	NA				Yes	Yes	Yes	NA	NA	CKD, died at 58 years due to kidney failure	2023	Schigt et al. (2023)
34	NA	NA	NA					Yes	Yes	NA	NA	CKD	2023	Schigt et al. (2023)
35	NA	NA	NA	NA	NA	NA	NA	Yes	Yes	NA	NA	CKD, deaf	2023	Schigt et al. (2023)
36		Yes			Yes	Yes		Yes	Yes	NA	NA	NA	2023	Schigt et al. (2023)
37	Yes			Yes	Yes	Yes		Yes	Yes	NA	NA	Labial fusion	2023	Schigt et al. (2023)
38	Yes	No	Yes	Yes	Yes	Yes	Yes	Yes	Yes	NA	NA	NA	2023	Yuan et al. (2023)
39	Yes	No	No	Yes	Yes	Yes	Yes	Yes	Yes	NA	No	No	2023	This study
40	Yes	Yes	Yes	Yes	No	No	No	Yes	No	Yes	Yes	No	2023	This study
41		Yes	Yes	Yes	Yes	Yes	Yes	Yes	No	No	No	No	2023	This study
42	No	Yes	No	Yes	No	No	No	NA	Yes	No	No	IUGR	2023	This study
43	No	No	No	Yes	No	No	No	NA	No	No	No	IUGR, ADHD	2023	This study
44	No	No	No	No	No	No	No	No	No	NA	No	No	2023	This study
45	No	No	No	NA	Yes	Yes	Yes	Yes	Yes	NA	No	No	2023	This study
46	No	No	No	Yes	NA	Yes	No	Yes	No	NA	No	Micropenis, microorchidism	2023	This study
Sum	11/28 (39.3%)	8/25 (32.0%)	8/26 (30.8%)	23/34 (67.6%)	25/36 (69.4%)	27/37 (73.0%)	12/34 (35.3%)	32/37 (86.5%)	34/41 (82.9%)	3/8 (37.5%)	4/11 (36.4%)	/	/	/

*Note*: An empty box indicates that the item is not mentioned in the original paper. Percentages calculated using the total number of patients for whom the features were measured/described (thus omitting missing data).

Abbreviations: AD, autosomal dominant; ADHD, attention‐deficit hyperactivity disorder; CKD, chronic kidney disease; d, days; F, female; GH, growth hormone; ID, intellectual disability; IUGR, intrauterine growth retardation; M, male; m, months; NA, not available; Ref., reference; Sum, summary; w.g., weeks of gestation; y, years.

### 3.1 Case presentation of our cohort

#### 3.1.1 Case 1 from family 1

**FIGURE 2:**
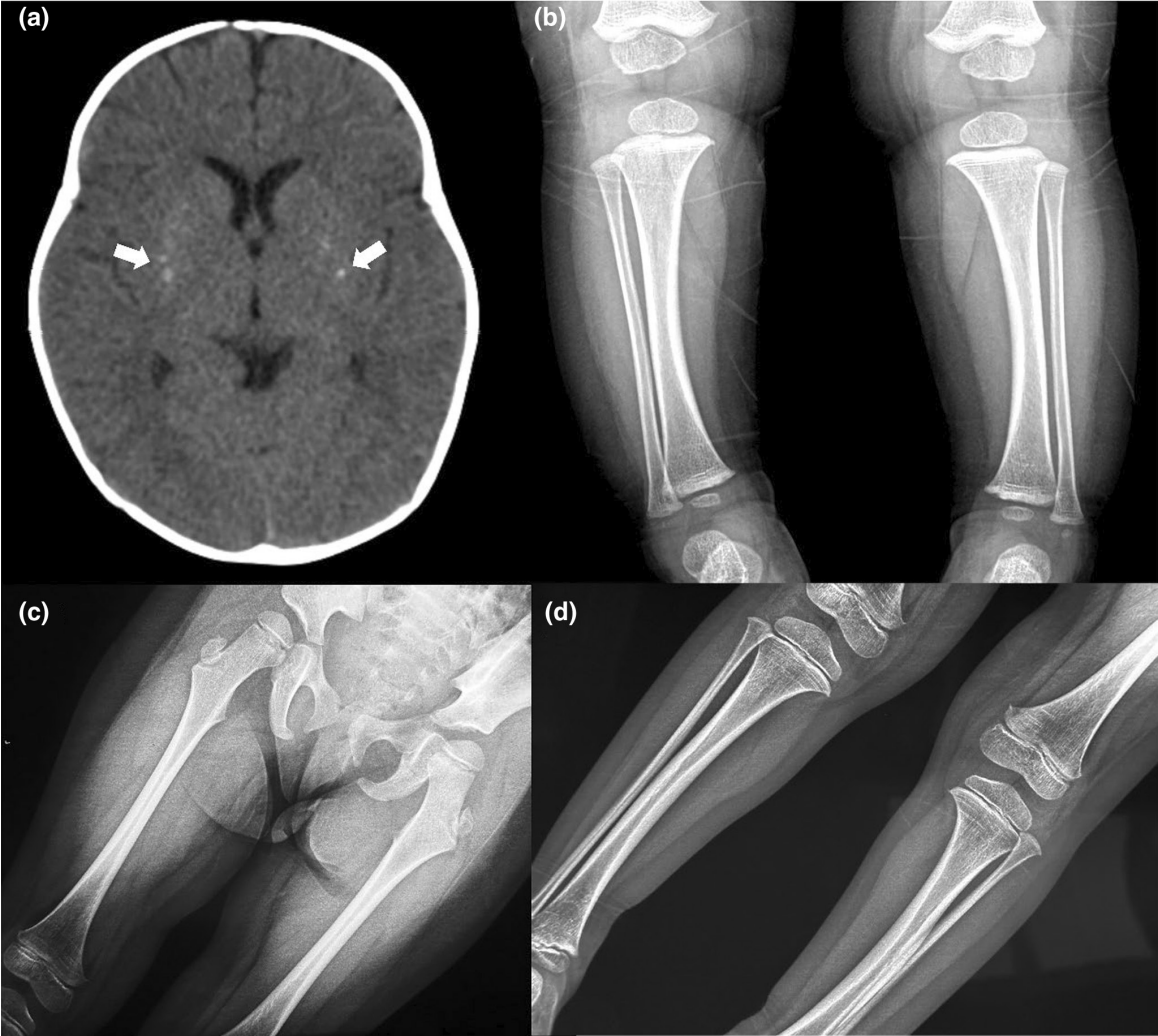
Radiographic studies of probands. (a) Brain computed tomography of F1‐II‐1. The arrowheads indicate calcifications in the basal ganglia. (b–d) X‐ray images show tubular long bones with cortical thickening and medullary stenosis in F1‐II‐1 (b) and F3‐II‐1 (c, d).

**FIGURE 3:**
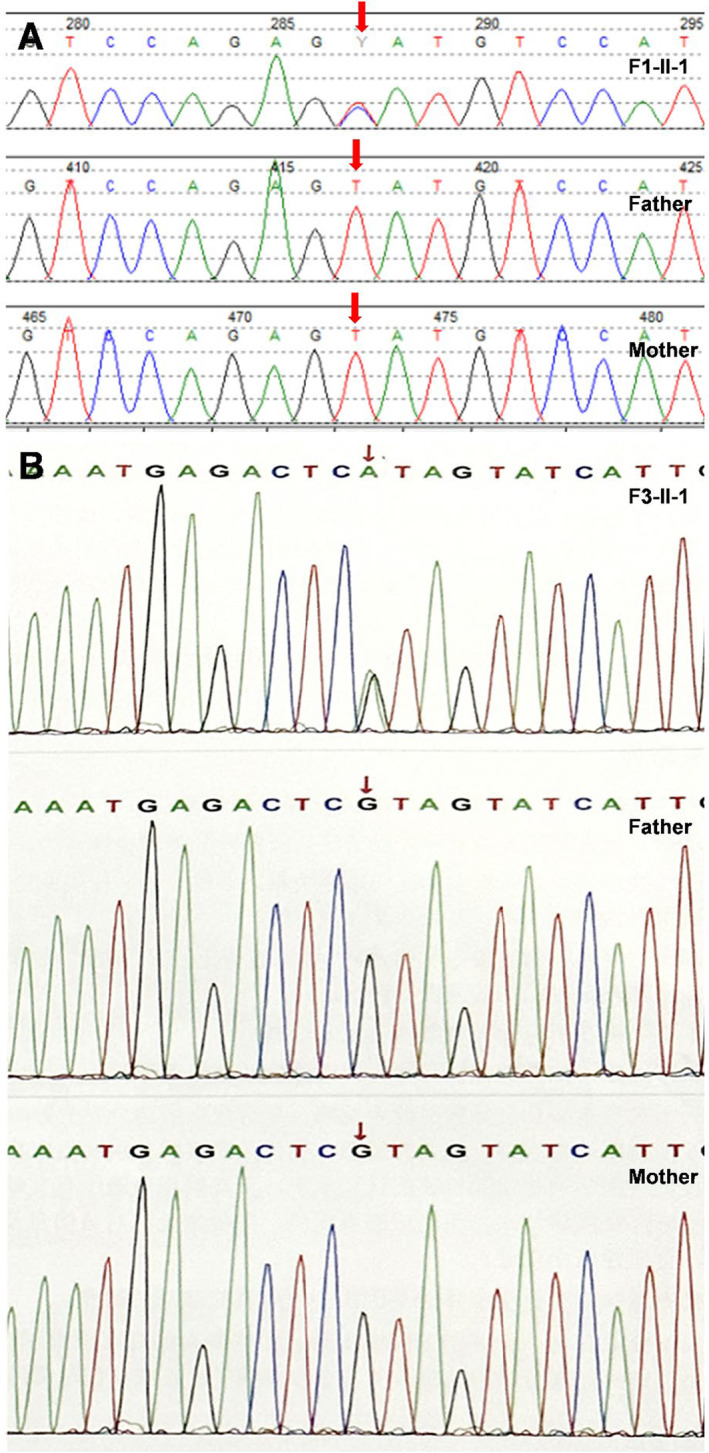
Electropherograms of Sanger sequencing of FAM111A variants in affected individuals and family members. (a) Heterozygous c.1531 T>C mutation in *FAM111A* (RefSeq: NM_001142519.1) in F1‐II‐1, but detected neither in her father nor in her mother. (b) Heterozygous c.1706 G>A mutation in *FAM111A* in F3‐II‐1, but detected neither in her father nor in her mother.

The girl (F1‐II‐1) was 2 years and 4 months old born at 39 weeks of gestation to nonconsanguineous, healthy Chinese parents. Ultrasound imaging showed shortened long bones during prenatal period. At birth, her length was 46 cm (−2.61 SD), her weight was 2700 g (−1.98 SD), and head circumference was 35.5 cm (1.4 SD) with large anterior fontanel. Her neonatal screening was unremarkable. At the age of 5 months, the patient was hospitalized due to bacterial meningitis. Hypocalcemia and hypoparathyroidism were detected. Her serum calcium (Ca), phosphorus (P), magnesium (Mg), and parathyroid hormone (PTH) levels were 1.9 mmol/L (reference range 2.10–2.80), 2.85 mmol/L (reference range 1.45–2.10), 0.55 mmol/L (reference range 0.66–1.05), and 4.8 pg/mL (reference range 12–88), respectively. Brain computed tomography (CT) revealed calcification in the basal ganglia (Figure 2a). Radiographs showed cortical thickening and medullary stenosis of lower limb bones (Figure 2b) at 9 months of age. At 15 months, she had an episode of generalized convulsions because of hypocalcemia. At this episode, her serum Ca, P, Mg and PTH levels were 1.31 mmol/L, 2.59 mmol/L, 0.59 mmol/L and 10.8 pg/mL, respectively. In follow‐up, the girl's height was 77 cm (−3.21 SD), and weight was 11 kg (−1.42 SD) at the age of 2 years and 4 months. Her anterior fontanelle remains open, and she has defective dentition and microdontia. Besides prominent forehead and depressed nasal root, no other dysmorphic features were observed. She was put on calcium, magnesium and vitamin D preparations and calcitriol, and did not have further seizure or signs of hypocalcemia. Whole exome sequencing (WES) revealed a missense variant in exon 4 of *FAM111A* (c.1531 T>C) resulting in amino acid substitution (p.Tyr511His). None of her parents had this alteration, indicating that the variant occurred de novo (Figure 3a).

#### 3.1.2 Case 2 from family 2

Patient 2 (F2‐II‐1), a 6‐year‐old girl, is the first and only child of healthy, nonconsanguineous parents. Intrauterine growth retardation and shortened long bones were noticed in the fetal period. She was born at 39 weeks of gestation, weighing 2500 g (−2.31 SD), measuring 46 cm (−2.61 SD), with head circumference of 34 cm (0.1 SD). The child had bilateral high hyperopia (a grade of +12 diopters) and amblyopia with tortuous retinal vessels, and needed corrective glasses since the age of 5. Serious dental caries, oligodontia and delayed eruption of the first deciduous tooth were noticed in the patient. She had only one hospitalization due to herpangina at 5 years old, and there have never been seizures or convulsions. The intact PTH level was less than 0.32 pmol/L (reference range 1.3–6.8). Basal serum Ca, P, Mg and insulin‐like growth factor (IGF‐I) levels were within normal limits. Cranial magnetic resonance imaging (MRI) and radiographic evaluation of long bones found no abnormalities. The growth hormone stimulation tests of arginine and levodopa showed a peak of 2.65 ng/mL and 0.44 ng/mL, respectively, suggestive of GH deficiency. GH treatment was commenced at 4 years and 2 months until the time this manuscript was written, with a growth velocity of 8 cm/year. Good compliance with administration of injections was maintained and no side effects occurred. At 6 years of age, her height was 89 cm (−5.86 SD), weight was 11 kg (−6.35 SD), and head circumference was 47 cm (−3.1 SD). Physical examination showed prominent forehead, small appearing eyes and flat nasal root. The anterior fontanelle remains open. She had normal intelligence for her age. WES was performed due to her short stature, severe visual impairment and defective dentition, and a de novo heterozygous c.1706 G>A (p.Arg569His) pathogenic variant in *FAM111A* was detected.

#### 3.1.3 Case 3 from family 3

The 5‐year‐and‐9‐month‐old girl (F3‐II‐1) was born as the first child of healthy, nonconsanguineous parents at 39 weeks of gestation. Pregnancy was uneventful except for comparatively shorter femur of the fetus discovered by ultrasound. Her birth weight and length were 2850 g (−0.9 SD) and 49 cm (−0.1 SD), and head circumference was 34 cm (0.1 SD). She was diagnosed with hypermetropia (a grade of +6.5 diopters on both eyes), amblyopia and pseudopapilledema, and has been wearing glasses since she was 4 years old. The patient presented with several decayed teeth, and CT revealed partial loss of permanent dental germ at 4 years and 6 months. She had only one hospitalization due to exanthema subitum at 1 year and 8 months, and there have never been seizures or convulsions. She has been short in stature since she was 1 month old, and her height 59 cm at 6 months, 67 cm at 1 year, 75 cm at 2 years, 81 cm at 3 years, 85 cm at 4 years, 90 cm at 4 years and 8 months (all < −3 SD). Growth hormone releasing test by insulin showed a normal GH reserve with a peak of 11.8 ng/mL at 4.8 years of age. Hyperphosphatemia and hypoparathyroidism were detected. Her serum P and PTH levels were 1.69 mmol/L (reference range 0.85–1.51) and 4.95 pg/mL (reference range 15–65). However, the serum Ca level was always within the normal range. Cranial MRI revealed fine calcification (punctate calcified foci) in right brain parenchyma. X‐ray of bilateral femora and tibiofibular showed cortical thickening and medullary stenosis, and delay of bone age (Figure 2c,d). On physical examination, performed at 5 years and 9 months of age, the stature was 95 cm (−3.99 SD), weight of 13.5 kg (−3.35 SD), and head circumference of 49 cm (−1.28 SD). Her anterior fontanelle remains open. Facial dysmorphisms included small eyes, high arched palate and micrognathia. Genetic investigations with exome sequencing followed by confirmation with Sanger sequencing identified a heterozygous missense variant in *FAM111A* gene: c.1706 G>A (p.Arg569His), which was not detected in her parents (Figure 3b).

#### 3.1.4 Case 4 from family 4

Patient 4 (F4‐II‐1) was the elder one of a pair of female twins, conceived naturally to nonconsanguineous Chinese parents. Her mother suffered from gestational diabetes mellitus and needed an insulin injection at 7 months of gestation. The fetus was found to have mild shortening of the extremities by prenatal sonography. She was born at 36 weeks and 4 days of gestation with birth weight of 2180 g (−2.82 SD). Hypocalcemia was detected on day 3 after birth with serum calcium level of 0.4–0.8 mmol/L, and intravenous calcium was administrated. Later oral Ca supplement was started, and her calcium level was stabilized. She never had hypocalcemic seizures. She had hypermetropia and astigmatism without cataract and needed corrective glasses since the age of 2. She had severe dental caries with the amount of 15, and the interdental space was large. On physical examination at 6 years and 6 months, her height was 94 cm (−5.25 SD), and weight was 11 kg (−7 SD). Dysmorphic features included sparse hair, prominent forehead, small appearing eyes, depressed nasal bridge, low‐set ears, micrognathia, small hands and feet. Her anterior fontanelle remains open. Dual‐energy X‐ray absorptiometry showed that the total bone mineral density T‐score was −8.0, indicating low bone density. Radiographs showed slender diaphyses of bilateral femora, without cortical thickening or medullary stenosis. Head MRI detected no calcification. Growth hormone releasing test showed a normal GH reserve at the age of 5 years and 7 months.

#### 3.1.5 Case 5 from family 4

Patient 5 (F4‐II‐2) was the younger twin sister of patient 4. Her birth weight was 1800 g (−3.7 SD). Short limbs were also noticed in the foetal period. She presented with visual impairment, especially in her right eye. Ophthalmological evaluation disclosed pseudopapilledema, and she has been treated with acetazolamide tablets twice a day since the age of 2. She also had bilateral hyperopia and astigmatism without cataract (a grade of +4 diopters). She was diagnosed with attention‐deficit hyperactivity disorder at 5 years of age, and has been under intervention. The girl had liver dysfunction, and ultrasound scan of liver showed abnormal inferior vena cava. On physical examination at 6.5 years of age, her height was 84 cm (−7.91 SD), and weight was 10 kg (−8.59 SD). Her anterior fontanelle remains open. Dysmorphic features consisted of microcephaly, sparse hair, prominent forehead, open metopic ridge, small eyes, depressed nasal bridge, low‐set ears, micrognathia and micromelia. Radiographs showed slender diaphyses with low bone density but no cortical thickening or medullary stenosis. Head MRI detected no calcification. Growth hormone releasing test showed the GH reserve in the lower limit. Her serum Ca, P, Mg and thyroid hormone levels were within normal range.

#### 3.1.6 Case 6 from family 4

The twins' father (F4‐I‐1) presented with proportionate short stature with height of 145 cm (−4.39 SD) (the grandfather's height 175 cm (−0.23 SD), the grandmother's height 168 cm (0.73 SD)). Besides, he had hypermetropia and astigmatism (a grade of +8 diopters) and required corrective glasses. He had no hypocalcemia, hyperphosphatemia or convulsions. Zygosity testing confirmed that patient 4 and patient 5 were monozygotic twins. Copy number variant (CNV) detection was performed in the twins, and there was no indication of a clinically relevant deletion or duplication of three or more exons in the genes evaluated. Clinical exome sequence analysis of this family revealed a heterozygous c. 1706 G>A (p.Arg569His) variant in the twins and their father. Their mother did not harbor this variant in the *FAM111A* gene, indicating a father‐to‐daughter transmission.

#### 3.1.7 Case 7 from family 5

The 11‐month‐old infant (F5‐II‐2) is the second child of healthy, nonconsanguineous Chinese parents. The first child of this family is a 4‐year‐old health girl. Her mother had gestational diabetes mellitus and controlled the blood glucose level by diet. She was born at 37 weeks of gestation with birth weight of 2930 g (−0.7 SD), length of 50 cm (0.5 SD), and head circumference of 31 cm (−2.4 SD). The proband came to medical attention in the neonatal period due to generalized convulsions secondary to low serum calcium and magnesium levels. Her serum Ca level was 1.26–1.4 mmol/L (reference range 1.9 to 2.6) and Mg was 0.46–0.58 mmol/L (reference range 0.62–0.91), and P level was 3.01–3.67 mmol/L (reference range 1.4–2.5). The parathyroid hormone value was 1.5–2.0 pmol/L (reference range 1.6–6.9). Kidney CT showed that the density of the renal medullary was slightly increased. When the proband was 3 months of age, brain CT revealed multiple fine calcifications in the basal ganglia. The skeletal scan showed cortical thickening and medullary stenosis of long bones. The calcium level was not stable, and the oral calcium gluconate treatment was continued intermittently. At 7 months of age, she was able to maintain her calcium level off replacement. Dysmorphic face (frontal bossing, small appearing eyes, and depressed nasal bridge) and open anterior fontanel (2.5 × 2.5 cm) were noted at follow‐up assessment. At the age of 11 months, her body length, body weight and head circumferences were 66 cm (−2.7 SD), 6.8 kg (−2.1 SD) and 44 cm (−0.4 SD), respectively. WES analysis of this family showed a heterozygous missense variant in *FAM111A* (c.1706 G>A), resulting in amino acid substitution (p.Arg569His). None of the parents and her elder sister had this alteration, indicating that the variant occurred de novo.

#### 3.1.8 Case 8 from family 6

The male patient (F6‐II‐1) was born as the first child of healthy, nonconsanguineous parents at 39 weeks of gestation, and shortened long bones were revealed by ultrasound imaging during the prenatal period. At birth, the patient weighed 3100 g (−0.5 SD) and measured 46 cm (−2.1 SD) in length. His neonatal screening was unremarkable. He had only one hospitalization due to pneumonia, and there have never been seizures or convulsions. Basal serum Ca, P, Vit D, alkaline phosphatase (ALP) and insulin‐like growth factor (IGF‐I) levels were within normal limits. However, the PTH level was low (8.0 pg/mL, reference range 10–69). Radiographs showed slender diaphyses of tubular bones, with medullary stenosis. Brain MRI performed at the age of 3 years found no abnormalities. On last physical examination, performed at 4 years of age, the stature was 84 cm (−4.44 SD), and weight was 12 kg (−2.96 SD). His anterior fontanelle remains open. External genitalia was male, but micropenis and microorchidism were notable. Dental and ocular assessment did not reveal any abnormalities. His neurodevelopment was normal. Trio‐WES detected a heterozygous c.1706 G>A (p.Arg569His) variant in *FAM111A* in this proband, but not in his parents.

### 3.2 Clinical characteristics of all patients

We have summarized the clinical characteristics and molecular findings of all reported and genetically confirmed cases of KCS2 in Table 2. Among these patients, 25 were females and 20 were males, and one was of undetermined sex because of the insufficient raw information. The male‐to‐female ratio was 1:1.25, and no apparent sex predominance was observed. A total of 10 variant types have been discovered, 32 patients (69.6%) carried the FAM111A Arg569His common variant, 3 siblings carried Glu535Gly, 2 patients carried Tyr511His, and a pair of twins carried Ser541Pro missense changes, while the others carried a single mutation type.

Together with previously reported KCS2 patients (Table 2), short stature represented the most common phenotype and was present in all individuals of our cohort and in 40 of 42 patients. Biochemical investigations demonstrated that the majority of patients (≥80%) had hypoparathyroidism and hypocalcemia. Ocular defects were found in 30 of 40 individuals (75%). Hypermetropia was the most common refractive anomaly, with 22 of 37 patients (60%) suffering from it. Pseudopapilledema, which was supposed to result from electrolyte disturbance (Cheng et al., 2021), was detected in 5 of 37 individuals (14%). Cataract was found in only one 40‐year‐old female with Arg569His variant. Myopia was also found in only one 12‐year‐old female with Arg569His variant. Although they were visually impaired, none had visual loss or blindness. Patients with KCS frequently had dental problems, including delayed dental eruption, dental caries, premature shedding of permanent dentition, enamel defects, oligodontia and microdontia (Moussaid et al., 2012). Dental anomalies were present in 21 of 29 KCS2 patients (72%) for whom information has been provided, as 39% of these patients had defective dentition, 32% had dental caries and 31% had oligodontia. But the exact prevalence of ocular and dental abnormalities was likely to be underestimated because some phenotypes such as hypermetropia and dental caries would arise at an older age, and some individuals had not reached the corresponding age, take our 11‐month‐old F5‐II‐2, for example. Prominent forehead was the consistently reported facial feature and present in 25 out of 34 patients (74%). Abnormal skeletal features were identified as prominent phenotypes, with 27 of 37 individuals (73%) exhibiting medullary stenosis of the tubular bones, 25 of 36 individuals (69%) exhibiting cortical thickening of tubular bones, and 23 of 34 individuals (68%) exhibiting delayed closure of anterior fontanelle. Up to 70% of patients have suffered seizures or spasms secondary to hypocalcemia, but there existed three patients not experiencing seizures despite their low serum calcium.

In this analysis, we also compared the phenotypes of two groups of KCS2 patients, based on the locations of their *FAM111A* variants: individuals with c.1706 G>A variant (*N* = 32) and individuals with any variants other than c.1706 G>A (*N* = 14). The two groups were compared in the main clinical characteristics including skeletal, ocular, dental and endocrinological aspects (Table 3). However, there was no significant statistical difference in these features between the two groups.

**TABLE 3:** Relationship between location of *FAM111A* gene variant and phenotypic consequences in patients with KCS2.

Phenotypic consequences	c.1706G>A variant (*N* = 32)	Other variants (*N* = 14)	*Z*/*χ* ^2^	*p*‐Value
Gender (Female/Male)	20/11	5/9	3.240	0.072
Diagnostic age (years, mean)	10.69	19.32	−1.187	0.254

	Number/total, %	Number/total, %	*Z*/*χ* ^2^	*p*‐Value
Short stature	29/30, 96.67%	11/12, 91.67%	/	0.495
Intellectual disability	3/31, 9.68%	2/12, 16.67%	0.012	0.912
Seizures/spasms	15/24, 62.5%	8/9, 88.89%	/	0.217
Ocular defects	24/31, 77.42%	6/9, 66.67%	0.048	0.827
Hypermetropia	19/28, 67.86%	3/9, 33.33%	/	0.118
(pseudo‐)papilledema	5/28, 17.86%	0/9, 0.00%	/	0.307
Dental problems	16/24, 66.67%	5/5, 100.00%	/	0.283
Defective dentition	9/23, 39.13%	2/5, 40.00%	/	1.000
Dental caries	7/21, 33.33%	1/4, 25.00%	/	1.000
Oligodontia	8/22, 36.36%	0/4, 0.00%	/	0.277
Skeletal features	26/29, 89.66%	8/11, 72.73%	0.711	0.399
Cortical thickening	19/26, 73.08%	6/10, 60.00%	/	0.454
Medullary stenosis	22/28, 78.57%	5/9, 55.56%	/	0.215
Delayed closure of anterior fontanelle	15/23, 65.22%	8/11, 72.73%	/	1.000
Cerebral calcification	9/25, 36.00%	3/9, 33.33%	/	1.000
Endocrinological abnormalities	27/31, 87.10%	12/12, 100.00%	0.520	0.471
Hypoparathyroidism	21/26, 80.77%	11/11, 100.00%	/	0.295
Hypocalcemia	23/30, 80.00%	11/11, 100.00%	1.666	0.197
Growth hormone deficiency	3/7, 42.86%	0/2, 0.00%	/	0.500

## 4 DISCUSSION

In this study, we present eight genetically confirmed KCS2 cases from six families. We identified the recurrent heterozygous missense FAM111A mutation, Arg569His, in seven patients (87.5%). Beyond that, Tyr511His was detected in one sporadic case (12.5%). Our patient cohort contributes to establishing typical features of KCS2 and confirms that Arg569His is the hot spot variant. In addition, we reviewed all the literature on KCS2, including 18 papers in English and 2 papers in Chinese. Only 46 cases of KCS2, including the current cases, have been reported, suggesting the extremely rare disorder.

Our analysis indicated that short stature was the most common phenotype, occurring in 95% of patients with KCS2. Skeletal features such as cortical thickening and medullary stenosis of tubular bones, endocrinological abnormalities including hypoparathyroidism and hypocalcemia, represent the typical phenotypes for KCS2. Besides, ocular and dental problems including hypermetropia, pseudopapilledema, defective dentition and dental caries, as well as seizures secondary to hypocalcemia, can also be instructive for this disorder. The mean diagnostic age was 13.2 years (range 20 weeks of gestation to 66 years), indicating the delayed diagnosis in patients with KCS2. It is possible that these patients could be misdiagnosed because phenotypes associated with hypoparathyroidism (Gafni & Collins, 2019) also appeared in other conditions such as 22q11.2 deletion syndrome (McDonald‐McGinn et al., 2015) and HDR syndrome (Van Esch & Devriendt, 2001). Molecular genetic studies have discovered at least 10 candidate genes related to hypoparathyroidism (Fukumoto et al., 2008; Nesbit et al., 2013; Wang et al., 2019). Therefore, identification of the above typical phenotypes of KCS2 would help physicians make the appropriate diagnosis, so as to facilitate early diagnosis and timely treatment. Additionally, gene detection is also essential to the definitive diagnosis.

We compared the phenotypes of patients with hotspot mutation (c.1706G>A) to those with other pathogenic variants but found no significant difference between the two groups. This result is in line with the finding of Schigt et al. (2023), suggesting that the genotype and phenotype are not closely related within KCS2. However, as more cases are genetically investigated, we will gain a better understanding of the relation between genotype and phenotype.

Despite the autosomal dominant inherited pattern of KCS2, de novo variants occurred in the majority of cases (31/39, 79.5%). There have been few parent‐to‐child transmissions in KCS2, even in KCS. The initial paper on KCS described a mother‐to‐son transmission by Kenny and Linarelli (1966). Later, Nikkel et al. (2014) reported the first molecularly confirmed instance of mother‐to‐child transmission of KCS2. More recently, Schigt et al. reported the paternal inheritance of KCS2, a male patient with his son and two daughters (Schigt et al., 2023). However, the father has not undergone gene testing, while his three children were investigated with WES and found to carry a novel heterozygous variant c.1604A>G. We present the first genetically confirmed instance of father‐to‐daughter transmission of KCS2 (Family 4), which demonstrates an equal sex ratio of affected individuals (Nikkel et al., 2014), consistent with an autosomal dominant inheritance.

The boy in our cohort had micropenis and microorchidism, and we also found five other male patients reported with micropenis and (or) microorchidism in the literature. The mechanism for micropenis and microorchidism has not been determined, and the laboratorial findings were inconsistent. Hoffman et al. (1998) investigated two adolescent boys with KCS and microorchidism. Endocrine studies on one patient revealed elevated level of serum follicle‐stimulating hormone, while testicular histology in another detected leydig cell hyperplasia. A pair of adult twins with KCS2 and microorchidism showed normal sexual hormone profile and normal puberty tempo (Cheng et al., 2021). Gonadal axis analysis on an 18‐year‐old male with micropenis and microorchidism revealed hypergonadotropic hypogonadism (Cavole et al., 2020). These data suggest that FAM111A may also play a role in the development of male genitalia. Male patients with KCS2 were believed to be subfertility before because there had been no reports of a father‐to‐child transmission and due to several reports of male with micropenis and (or) microorchidism (Cheng et al., 2021; Hoffman et al., 1998). Nevertheless, our first genetically confirmed paternal inheritance refutes this opinion.

Intriguingly, recent reports and our own observations have challenged some conventional distinctions. Although reduced PTH with hypocalcemia was observed in over 80% of individuals (Table 2), representing the typical sign of KCS2, patients with normal PTH have also been described (Deconte et al., 2020; Guo et al., 2014; Lang et al., 2021). Less than half of our cohort has hypocalcemia, and three patients with low PTH levels also had normal calcium levels. Likewise, cortical thickening or medullary stenosis of the long bones was not observed in half of our patients and several reports (Deconte et al., 2020; Schigt et al., 2023). Previous studies showed that patients with KCS2 did not exhibit prenatal growth restriction and suggested that FAM111A is predominantly involved in postnatal growth (Guo et al., 2014). However, intrauterine growth retardation, which is defined as low birth weight of ≤ −2 SD (Lee et al., 2003), was observed in three patients of our cohort and two reported cases (Nikkel et al., 2014; Schigt et al., 2023). Furthermore, ultrasound imaging in the gestation period detected shortened long bones of limbs in six cases of our cohort. This finding is consistent with the study that PTH‐deficient mice showed reduced metaphyseal osteoblasts and trabecular bone, increased bone mineralization and shortened long bones (Miao et al., 2002). Our results indicate that PTH is necessary for normal foetal skeletal development. However, this recurrent condition has not been emphasized in previous reports. Although patients with KCS2 were traditionally considered to have normal intelligence, intellectual disability was noted in five patients in three recent publications (Cavole et al., 2020; Deconte et al., 2020; Schigt et al., 2023). Therefore, further case accumulation and investigations are needed, as well as a comprehensive overview of symptoms and laboratory findings, in order to get a better understanding of the presentations of this rare disorder.

FAM111A‐related disorders include KCS2 and OCS. While KCS2 shows milder phenotypes with short stature, impaired skeletal development, recurrent hypoparathyroidism and hypocalcemia which requires supplement of calcium and vitamin D (Isojima et al., 2014; Unger et al., 2013), patients with OCS are usually stillborn or die shortly after birth (Müller et al., 2021; Pemberton et al., 2020). To date, no consensus clinical diagnostic criteria for FAM111A‐related disorders have been published. Most variants reported in the literature are clustered near the C‐terminus of FAM111A protein, and a definite genotype–phenotype relationship which explains the different severities of OCS and KCS has not been established yet. Rosato et al. (2022) reviewed the previous reported OCS cases, and found that intrauterine growth retardation, limb undergrowth, cloverleaf‐shaped skull, slender long bones with diaphyseal stenosis and metaphyseal flaring, splenic hypoplasia or aplasia were the most distinctive characteristics of OCS. A comparison of phenotypes of 22 KCS2 and 13 OCS individuals showed that intrauterine growth deficiency, cloverleaf‐shaped skull, decreased skull ossification, slender long bones and flared metaphyses were reported in almost all individuals/fetuses with OCS (Cheng et al., 1993). However, these phenotypes were rarely reported in individuals with KCS2. Anomalies such as cloverleaf‐shaped skull, flared metaphyses and fractures were not present in KCS2 cases (Isojima et al., 2014; Schigt et al., 2023). Likewise, ophthalmologic problems including refractive errors and (pseudo‐)papilledema, dental abnormalities including defective dentition and dental caries, have not been reported in OCS individuals (Cheng et al., 1993; Eren et al., 2021; Unger et al., 2013).

**FIGURE 4:**
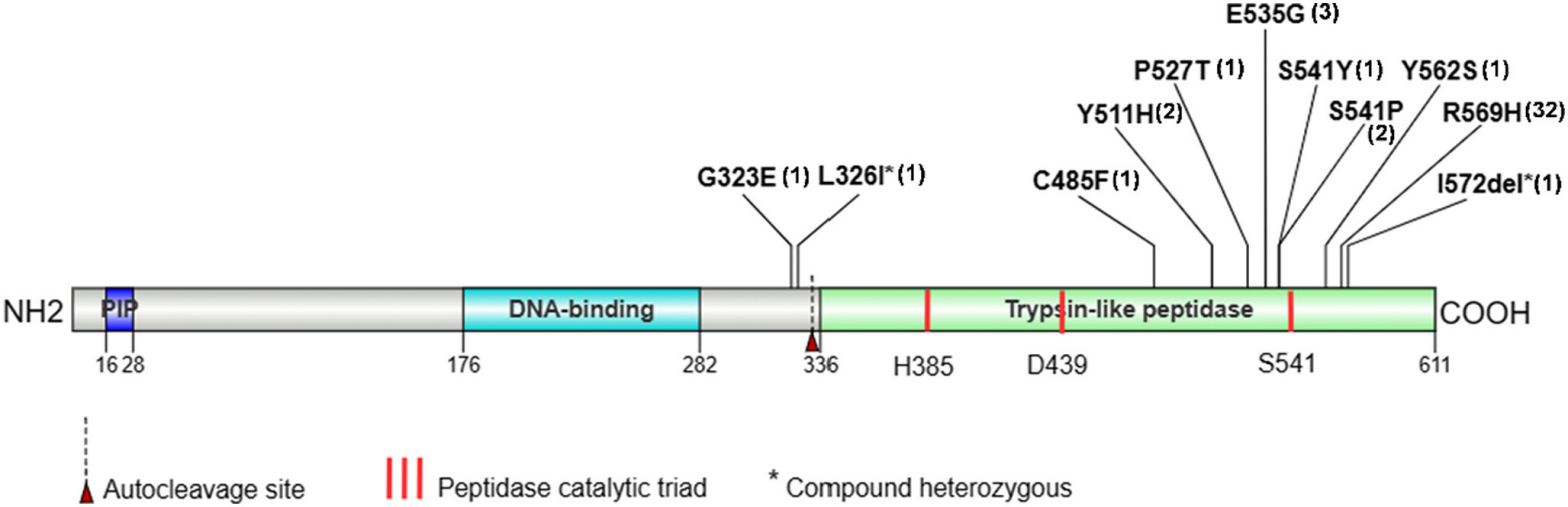
Scheme of the FAM111A protein and known (likely) pathogenic variants associated with Kenny–Caffey Syndrome Type 2 (KCS2) (the digit in the parentheses represents the number of cases of this variant type). KCS2 variants in FAM111A show clustering in and around the serine peptidase domain and autocleavage site. The domain in green has homology to trypsin‐like peptidases, including a conserved putative catalytic triad purportedly composed of Ser541/His385/Asp439 (red bars). PIP, PCNA‐interacting protein. *Compound heterozygous variants that were found together in one KSC2 patient.

*FAM111A* encodes a protein of 611 amino acids that has a carboxy‐terminal region homologous to trypsin‐like peptidases and a conserved catalytic triad specific to such peptidases (Figure 4). Transcriptional expression of FAM111A is ubiquitous (Isojima et al., 2014), and it is constitutively expressed in parathyroid and bone (Eren et al., 2021; Unger et al., 2013). The native functions of FAM111A variants and the pathogenesis of KCS2 are not completely understood, and still being investigated. All the variants identified in the KCS2 to date lead to substitutions or deletions of single amino acid, but do not result in frameshifts or premature terminations (Figure 4), indicating that haploinsufficiency is not sufficient to cause KCS2 (Unger et al., 2013). R569H, a hotspot variant, was detected in seven of eight individuals of our cohort. Molecular modelling has shown that residues of KCS2 are not located close to the putative active site but rather clustered on or near the outer surface of the protein (Unger et al., 2013). On account of the position of residues affected by KCS2, the variants were predicted to weaken or prevent intermolecular interactions with unidentified partners (Isojima et al., 2014; Unger et al., 2013). The model is consistent with the observation that patient‐associated *FAM111A* point mutations cause multisystem disorders via a common gain‐of‐function mechanism, which hyperactivates its intrinsic proteolytic activity to suppress DNA replication and transcription (Hoffmann et al., 2020). Most variants occupy on the C‐terminus of the FAM111A protein, which has homology to trypsin‐like peptidases. Other two variants, G323E and L326I, are located near the autocleavage site (Figure 4). It was speculated that the former resulted in constitutive protease activity while the latter might increase autocleavage activity of FAM111A protein, consequently leading to KCS2 (Kojima et al., 2020; Nie et al., 2021; Rosato et al., 2022). Nevertheless, little is known about the pathogenetic mechanism of this rare condition, future studies and accumulation of cases would be warranted in the future.

Patients with KCS2 have severe short stature (−3 to −10 SDS). At follow‐up, we noticed that parents were particularly concerned about the heights of their children, as well as the feasibility of GH therapy. GH tests have been conducted in four individuals in our cohort, of which three had normal overnight GH levels, but one (F2‐II‐1) showed GH deficiency. It was considered that the principal reason for short stature in KCS2 was not the perturbation of the GH‐IGF‐1 axis, but rather the skeletal defect which limits growth (Abraham et al., 2017). However, GH deficiency occurred in three of eight individuals (37.5%) in total for whom information has been provided (Isojima et al., 2014). Our patient with GH deficiency was started on GH therapy at 4 years and 2 months of age and had a growth response of 8 cm/y. A 12‐year‐old female was variably treated with GH and IGF‐1, and the height SDS improved from −5.99 to −3.38 (Guo et al., 2014). Another report of GH treatment on a 3‐year‐and‐3‐month‐old girl showed that her height has increased from −3.86SD to −3.18SD at 5.11 years of age (Abraham et al., 2017). Despite limited cases of GH therapy in patients with KCS2, reports such as these provide important information including GH levels and growth response of KCS2 with GH therapy. Further studies and collaboration between clinicians are needed to explore the long‐term effects of GH treatment, which would help guide clinical decisions on the use and doses of GH in KCS2.

In conclusion, we provided detailed characteristics of eight individuals with genetically confirmed KCS2, which has been the largest cohort of KCS2 so far in China. Furthermore, we reviewed all the literature about KCS2 and collected the phenotypes and genotypes of 46 patients in total. We confirmed that the c.1706G>A is the mutational hotspot in KCS2. While short stature and hormonal abnormalities such as hypoparathyroidism and hypocalcemia represent the most common phenotypes, the types and severity of the symptoms vary greatly in these patients. Questions regarding the pathogenic mechanism, genotype–phenotype correlation and treatment of this disorder remain unanswered. As more individuals are genetically investigated, we will get a better understanding of the molecular mechanism and overall clinical features of this rare condition.

## 5 AUTHOR CONTRIBUTIONS

Xuefei Chen and Chaochun Zou conceptualized and designed the study. Xuefei Chen collected the information of the patients, performed the literature search, analyzed data and drafted the initial manuscript. Chaochun Zou reviewed and revised the manuscript. All authors read, edited, and approved the final version of the manuscript.

## CONFLICT OF INTEREST STATEMENT

The authors declare that they have no conflict of interest.

## Data Availability

The authors confirm that the data supporting the findings of this study are available within the article and/or its supplementary materials.

## References

[mgg32433-bib-0001] Abraham, M. B. , Li, D. , Tang, D. , O'Connell, S. M. , McKenzie, F. , Lim, E. M. , Hakonarson, H. , Levine, M. A. , & Choong, C. S. (2017). Short stature and hypoparathyroidism in a child with Kenny‐Caffey syndrome type 2 due to a novel mutation in FAM111A gene. International Journal of Pediatric Endocrinology, 2017, 1. 28138333 10.1186/s13633-016-0041-7 PMC5264330

[mgg32433-bib-0002] Caffey, J. (1967). Congenital stenosis of medullary spaces in tubular bones and calvaria in two proportionate dwarfs–mother and son; coupled with transitory hypocalcemic tetany. The American Journal of Roentgenology, Radium Therapy, and Nuclear Medicine, 100, 1–11. 6023894 10.2214/ajr.100.1.1

[mgg32433-bib-0003] Cavole, T. R. , Perrone, E. , de Faria Soares, M. F. , Dias da Silva, M. R. , Maeda, S. S. , Lazaretti‐Castro, M. , & Alvarez Perez, A. B. (2020). Overlapping phenotype comprising Kenny‐Caffey type 2 and Sanjad‐Sakati syndromes: The first case report. American Journal of Medical Genetics. Part A, 182, 3029–3034. 33010201 10.1002/ajmg.a.61896

[mgg32433-bib-0004] Cheng, S. , Lo, I. F. M. , & Luk, H. M. (1993). FAM111A‐related skeletal Dysplasias. In M. P. Adam , J. Feldman , G. M. Mirzaa , R. A. Pagon , S. E. Wallace , L. J. H. Bean , K. W. Gripp , & A. Amemiya (Eds.), GeneReviews(®) University of Washington, Seattle Copyright © 1993–2024, University of Washington, Seattle. GeneReviews is a registered trademark of the University of Washington, Seattle. University of Washington, Seattle.

[mgg32433-bib-0005] Cheng, S. S. W. , Chan, P. K. J. , Luk, H. M. , Mok, M. T. , & Lo, I. F. M. (2021). Adult Chinese twins with Kenny‐Caffey syndrome type 2: A potential age‐dependent phenotype and review of literature'. American Journal of Medical Genetics. Part A, 185, 636–646. 33263187 10.1002/ajmg.a.61991

[mgg32433-bib-0006] Deconte, D. , Kreusch, T. C. , Salvaro, B. P. , Perin, W. F. , Ferreira, M. A. T. , Kopacek, C. , da Rosa, E. B. , Heringer, J. I. , Ligabue‐Braun, R. , Zen, P. R. G. , Rosa, R. F. M. , & Fiegenbaum, M. (2020). Ophthalmologic impairment and intellectual disability in a girl presenting Kenny‐Caffey syndrome type 2. Journal of Pediatric Genetics, 9, 263–269. 32765931 10.1055/s-0039-3401831 PMC7396477

[mgg32433-bib-0007] Elliott, A. M. , Wilcox, W. R. , Spear, G. S. , Field, F. M. , Steffensen, T. S. , Friedman, B. D. , Rimoin, D. L. , & Lachman, R. S. (2006). Osteocraniostenosis‐hypomineralized skull with gracile long bones and splenic hypoplasia. Four new cases with distinctive chondro‐osseous morphology. American Journal of Medical Genetics. Part A, 140, 1553–1563. 16770805 10.1002/ajmg.a.31326

[mgg32433-bib-0008] Eren, E. , Tezcan Unlu, H. , Ceylaner, S. , & Tarim, O. (2021). 'Compound heterozygous variants in FAM111A cause autosomal recessive Kenny‐Caffey syndrome type 2. Journal of Clinical Research in Pediatric Endocrinology, 15(1), 97–102. 34382758 10.4274/jcrpe.galenos.2021.2020.0315 PMC9976165

[mgg32433-bib-0009] Fanconi, S. , Fischer, J. A. , Wieland, P. , Atares, M. , Fanconi, A. , Giedion, A. , & Prader, A. (1986). Kenny syndrome: Evidence for idiopathic hypoparathyroidism in two patients and for abnormal parathyroid hormone in one. Journal of Pediatrics, 109, 469–475. 3746537 10.1016/s0022-3476(86)80120-2

[mgg32433-bib-0010] Fine, D. A. , Rozenblatt‐Rosen, O. , Padi, M. , Korkhin, A. , James, R. L. , Adelmant, G. , Yoon, R. , Guo, L. , Berrios, C. , Zhang, Y. , Calderwood, M. A. , Velmurgan, S. , Cheng, J. , Marto, J. A. , Hill, D. E. , Cusick, M. E. , Vidal, M. , Florens, L. , Washburn, M. P. , … DeCaprio, J. A. (2012). Identification of FAM111A as an SV40 host range restriction and adenovirus helper factor. PLoS Pathogens, 8, e1002949. 23093934 10.1371/journal.ppat.1002949 PMC3475652

[mgg32433-bib-0011] Franceschini, P. , Testa, A. , Bogetti, G. , Girardo, E. , Guala, A. , Lopez‐Bell, G. , Buzio, G. , Ferrario, E. , & Piccato, E. (1992). Kenny‐Caffey syndrome in two sibs born to consanguineous parents: Evidence for an autosomal recessive variant. American Journal of Medical Genetics, 42, 112–116. 1308349 10.1002/ajmg.1320420123

[mgg32433-bib-0012] Fukumoto, S. , Namba, N. , Ozono, K. , Yamauchi, M. , Sugimoto, T. , Michigami, T. , Tanaka, H. , Inoue, D. , Minagawa, M. , Endo, I. , & Matsumoto, T. (2008). Causes and differential diagnosis of hypocalcemia–recommendation proposed by expert panel supported by ministry of health, labour and welfare, Japan. Endocrine Journal, 55, 787–794. 18490837 10.1507/endocrj.k08e-076

[mgg32433-bib-0013] Gafni, R. I. , & Collins, M. T. (2019). Hypoparathyroidism. New England Journal of Medicine, 380, 1738–1747. 31042826 10.1056/NEJMcp1800213

[mgg32433-bib-0014] Guo, M. H. , Shen, Y. , Walvoord, E. C. , Miller, T. C. , Moon, J. E. , Hirschhorn, J. N. , & Dauber, A. (2014). Whole exome sequencing to identify genetic causes of short stature. Hormone Research in Pædiatrics, 82, 44–52. 10.1159/000360857 PMC4130218 24970356

[mgg32433-bib-0015] Hoffman, W. H. , Kovacs, K. , Li, S. , Kulharya, A. S. , Johnson, B. L. , Eidson, M. S. , & Cleveland, W. W. (1998). Kenny‐Caffey syndrome and microorchidism. American Journal of Medical Genetics, 80, 107–111. 9805124 10.1002/(sici)1096-8628(19981102)80:2<107::aid-ajmg3>3.0.co;2-v

[mgg32433-bib-0016] Hoffmann, S. , Pentakota, S. , Mund, A. , Haahr, P. , Coscia, F. , Gallo, M. , Mann, M. , Taylor, N. M. , & Mailand, N. (2020). FAM111 protease activity undermines cellular fitness and is amplified by gain‐of‐function mutations in human disease. EMBO Reports, 21, e50662. 32776417 10.15252/embr.202050662 PMC7534640

[mgg32433-bib-0017] Huang, Q. , Li, C. , Fan, X. , Xie, B. , Tan, L. , & Chen, Y. (2021). Kenny‐Caffey syndrome type 2: A case report and literature review. Chinese Journal of Birth Health & Heredity, 29, 1453–1457.

[mgg32433-bib-0018] Isojima, T. , Doi, K. , Mitsui, J. , Oda, Y. , Tokuhiro, E. , Yasoda, A. , Yorifuji, T. , Horikawa, R. , Yoshimura, J. , Ishiura, H. , Morishita, S. , Tsuji, S. , & Kitanaka, S. (2014). A recurrent de novo FAM111A mutation causes Kenny‐Caffey syndrome type 2. Journal of Bone and Mineral Research, 29, 992–998. 23996431 10.1002/jbmr.2091

[mgg32433-bib-0019] Kenny, F. M. , & Linarelli, L. (1966). Dwarfism and cortical thickening of tubular bones. Transient hypocalcemia in a mother and son. American Journal of Diseases of Children, 111, 201–207. 5322798 10.1001/archpedi.1966.02090050133013

[mgg32433-bib-0020] Kim, J. H. , Shin, Y. L. , Yang, S. , Cheon, C. K. , Cho, J. H. , Lee, B. H. , Kim, G. H. , Lee, J. O. , Seo, E. J. , Choi, J. H. , & Yoo, H. W. (2015). Diverse genetic aetiologies and clinical outcomes of paediatric hypoparathyroidism. Clinical Endocrinology, 83, 790–796. 26384470 10.1111/cen.12944

[mgg32433-bib-0021] Kojima, Y. , Machida, Y. , Palani, S. , Caulfield, T. R. , Radisky, E. S. , Kaufmann, S. H. , & Machida, Y. J. (2020). FAM111A protects replication forks from protein obstacles via its trypsin‐like domain. Nature Communications, 11, 1318. 10.1038/s41467-020-15170-7 PMC7067828 32165630

[mgg32433-bib-0022] Lang, E. , Koller, S. , Atac, D. , Pfäffli, O. A. , Hanson, J. V. M. , Feil, S. , Bähr, L. , Bahr, A. , Kottke, R. , Joset, P. , Fasler, K. , Barthelmes, D. , Steindl, K. , Konrad, D. , Wille, D. A. , Berger, W. , & Gerth‐Kahlert, C. (2021). Genotype‐phenotype spectrum in isolated and syndromic nanophthalmos. Acta Ophthalmologica, 99, e594–e607. 32996714 10.1111/aos.14615

[mgg32433-bib-0023] Lee, P. A. , Chernausek, S. D. , Hokken‐Koelega, A. C. , & Czernichow, P. (2003). International small for gestational age advisory board consensus development conference statement: Management of short children born small for gestational age, April 24‐October 1, 2001. Pediatrics, 111, 1253–1261. 12777538 10.1542/peds.111.6.1253

[mgg32433-bib-0024] McDonald‐McGinn, D. M. , Sullivan, K. E. , Marino, B. , Philip, N. , Swillen, A. , Vorstman, J. A. , Zackai, E. H. , Emanuel, B. S. , Vermeesch, J. R. , Morrow, B. E. , Scambler, P. J. , & Bassett, A. S. (2015). 22q11.2 deletion syndrome. Nature Reviews. Disease Primers, 1, 15071. 10.1038/nrdp.2015.71 PMC4900471 27189754

[mgg32433-bib-0025] Miao, D. , He, B. , Karaplis, A. C. , & Goltzman, D. (2002). Parathyroid hormone is essential for normal fetal bone formation. Journal of Clinical Investigation, 109, 1173–1182. 11994406 10.1172/JCI14817 PMC150965

[mgg32433-bib-0026] Moussaid, Y. , Griffiths, D. , Richard, B. , Dieux, A. , Lemerrer, M. , Léger, J. , Lacombe, D. , & Bailleul‐Forestier, I. (2012). Oral manifestations of patients with Kenny‐Caffey syndrome. European Journal of Medical Genetics, 55, 441–445. 22522175 10.1016/j.ejmg.2012.03.005

[mgg32433-bib-0027] Müller, R. , Steffensen, T. , Krstić, N. , & Cain, M. A. (2021). 'Report of a novel variant in the FAM111A gene in a fetus with multiple anomalies including gracile bones, hypoplastic spleen, and hypomineralized skull. American Journal of Medical Genetics. Part A, 185, 1903–1907. 33750016 10.1002/ajmg.a.62182

[mgg32433-bib-0028] Nesbit, M. A. , Hannan, F. M. , Howles, S. A. , Babinsky, V. N. , Head, R. A. , Cranston, T. , Rust, N. , Hobbs, M. R. , Heath, H., 3rd , & Thakker, R. V. (2013). Mutations affecting G‐protein subunit α11 in hypercalcemia and hypocalcemia. New England Journal of Medicine, 368, 2476–2486. 23802516 10.1056/NEJMoa1300253 PMC3773604

[mgg32433-bib-0029] Nie, M. , Oravcová, M. , Jami‐Alahmadi, Y. , Wohlschlegel, J. A. , Lazzerini‐Denchi, E. , & Boddy, M. N. (2021). FAM111A induces nuclear dysfunction in disease and viral restriction. EMBO Reports, 22, e50803. 33369867 10.15252/embr.202050803 PMC7857424

[mgg32433-bib-0030] Nikkel, S. M. , Ahmed, A. , Smith, A. , Marcadier, J. , Bulman, D. E. , & Boycott, K. M. (2014). Mother‐to‐daughter transmission of Kenny‐Caffey syndrome associated with the recurrent, dominant FAM111A mutation p.Arg569His. Clinical Genetics, 86, 394–395. 24635597 10.1111/cge.12290

[mgg32433-bib-0031] Ohmachi, Y. , Urai, S. , Bando, H. , Yokoi, J. , Yamamoto, M. , Kanie, K. , Motomura, Y. , Tsujimoto, Y. , Sasaki, Y. , Oi, Y. , Yamamoto, N. , Suzuki, M. , Shichi, H. , Iguchi, G. , Uehara, N. , Fukuoka, H. , & Ogawa, W. (2022). Case report: Late middle‐aged features of FAM111A variant, Kenny‐Caffey syndrome type 2‐suggestive symptoms during a long follow‐up. Frontiers in Endocrinology, 13, 1073173. 36686468 10.3389/fendo.2022.1073173 PMC9846794

[mgg32433-bib-0032] Parvari, R. , Hershkovitz, E. , Grossman, N. , Gorodischer, R. , Loeys, B. , Zecic, A. , Mortier, G. , Gregory, S. , Sharony, R. , Kambouris, M. , Sakati, N. , Meyer, B. F. , Al Aqeel, A. I. , Al Humaidan, A. K. , Al Zanhrani, F. , Al Swaid, A. , Al Othman, J. , Diaz, G. A. , Weiner, R. , … Gelb, B. D. (2002). Mutation of TBCE causes hypoparathyroidism‐retardation‐dysmorphism and autosomal recessive Kenny‐Caffey syndrome. Nature Genetics, 32, 448–452. 12389028 10.1038/ng1012

[mgg32433-bib-0033] Pemberton, L. , Barker, R. , Cockell, A. , Ramachandran, V. , Haworth, A. , & Homfray, T. (2020). Case report: Targeted whole exome sequencing enables the first prenatal diagnosis of the lethal skeletal dysplasia Osteocraniostenosis. BMC Medical Genetics, 21, 7. 31910817 10.1186/s12881-019-0939-z PMC6947839

[mgg32433-bib-0034] Rosato, S. , Unger, S. , Campos‐Xavier, B. , Caraffi, S. G. , Beltrami, L. , Pollazzon, M. , Ivanovski, I. , Castori, M. , Bonasoni, M. P. , Comitini, G. , Nikkels, P. G. J. , Lindstrom, K. , Umandap, C. , Superti‐Furga, A. , & Garavelli, L. (2022). Clinical and molecular diagnosis of Osteocraniostenosis in fetuses and newborns: Prenatal ultrasound, clinical, radiological and pathological features. Genes (Basel), 13, 261. 35205306 10.3390/genes13020261 PMC8871755

[mgg32433-bib-0035] Sabry, M. A. , Farag, T. I. , Shaltout, A. A. , Zaki, M. , Al‐Mazidi, Z. , Abulhassan, S. J. , Al‐Torki, N. , Quishawi, A. , & Al Awadi, S. A. (1999). Kenny‐Caffey syndrome: An Arab variant? Clinical Genetics, 55, 44–49. 10066031 10.1034/j.1399-0004.1999.550108.x

[mgg32433-bib-0036] Sabry, M. A. , Zaki, M. , Abul Hassan, S. J. , Ramadan, D. G. , Abdel Rasool, M. A. , al Awadi, S. A. , & al Saleh, Q. (1998). Kenny‐Caffey syndrome is part of the CATCH 22 haploinsufficiency cluster. Journal of Medical Genetics, 35, 31–36. 9475091 10.1136/jmg.35.1.31 PMC1051183

[mgg32433-bib-0037] Schigt, H. , Bald, M. , van der Eerden, B. C. J. , Gal, L. , Ilenwabor, B. P. , Konrad, M. , Levine, M. A. , Li, D. , Mache, C. J. , Mackin, S. , Perry, C. , Rios, F. J. , Schlingmann, K. P. , Storey, B. , Trapp, C. M. , Ajmh, V. , Zillikens, M. C. , Touyz, R. M. , Hoorn, E. J. , … de Baaij, J. H. F. (2023). Expanding the phenotypic spectrum of Kenny‐Caffey syndrome: A case series and systematic literature review. Journal of Clinical Endocrinology and Metabolism, 108, e754–e768. 36916904 10.1210/clinem/dgad147 PMC10438882

[mgg32433-bib-0038] Turner, A. E. , Abu‐Ghname, A. , Davis, M. J. , Shih, L. , Volk, A. S. , Streff, H. , & Buchanan, E. P. (2020). Kenny‐Caffey syndrome type 2: A unique presentation and craniofacial analysis. The Journal of Craniofacial Surgery, 31, e471–e475. 32310878 10.1097/SCS.0000000000006439

[mgg32433-bib-0039] Unger, S. , Górna, M. W. , Le Béchec, A. , Do Vale‐Pereira, S. , Bedeschi, M. F. , Geiberger, S. , Grigelioniene, G. , Horemuzova, E. , Lalatta, F. , Lausch, E. , Magnani, C. , Nampoothiri, S. , Nishimura, G. , Petrella, D. , Rojas‐Ringeling, F. , Utsunomiya, A. , Zabel, B. , Pradervand, S. , Harshman, K. , … Superti‐Furga, A. (2013). FAM111A mutations result in hypoparathyroidism and impaired skeletal development. American Journal of Human Genetics, 92, 990–995. 23684011 10.1016/j.ajhg.2013.04.020 PMC3675238

[mgg32433-bib-0040] Van Esch, H. , & Devriendt, K. (2001). Transcription factor GATA3 and the human HDR syndrome. Cellular and Molecular Life Sciences, 58, 1296–1300. 11577985 10.1007/PL00000940 PMC11337399

[mgg32433-bib-0041] Verloes, A. , Narcy, F. , Grattagliano, B. , Delezoide, A. L. , Guibaud, P. , Schaaps, J. P. , Le Merrer, M. , & Maroteaux, P. (1994). Osteocraniostenosis. Journal of Medical Genetics, 31, 772–778. 7837254 10.1136/jmg.31.10.772 PMC1050124

[mgg32433-bib-0042] Wang, Y. , Nie, M. , Wang, O. , Li, Y. , Jiang, Y. , Li, M. , Xia, W. , & Xing, X. (2019). Genetic screening in a large Chinese cohort of childhood onset hypoparathyroidism by next‐generation sequencing combined with TBX1‐MLPA. Journal of Bone and Mineral Research, 34, 2254–2263. 31433868 10.1002/jbmr.3854

[mgg32433-bib-0043] Xu, N. , Wang, Y. , Tingting, Y. , Chen, Y. , Li, X. , Wang, J. , Wang, X. , & Li, J. (2019). Kenny‐Caffey syndrome caused by mutation of FAM111A gene: A case report and literature review. Journal of Clinical Pediatrics, 37, 369–372.

[mgg32433-bib-0044] Yerawar, C. , Kabde, A. , & Deokar, P. (2021). Kenny‐Caffey syndrome type 2. QJM, 114, 267–269. 32428224 10.1093/qjmed/hcaa175

[mgg32433-bib-0045] Yuan, N. , Lu, L. , Xing, X. P. , Wang, O. , Jiang, Y. , Wu, J. , He, M. H. , Wang, X. J. , & Cao, L. W. (2023). Clinical and genetic features of Kenny‐Caffey syndrome type 2 with multiple electrolyte disturbances: A case report. World Journal of Clinical Cases, 11, 2290–2300. 37122511 10.12998/wjcc.v11.i10.2290 PMC10131010

